# Revealing an initiation inhibition of RCA and its application in nucleic acid detection: Initiation inhibition of RCA to detect nucleic acids

**DOI:** 10.3724/abbs.2023070

**Published:** 2023-04-20

**Authors:** Qian Zhuang, Meiqiong Ge, Xiaodong Yu, Jing Chai, Lu Zhang, Suming Tang, Hua Wei, Jinxin Zhang, Guojie Zhao

**Affiliations:** 1 Department of Biochemistry and Molecular Biology China Medical University Shenyang 110122 China; 2 Department of Pediatrics Shengjing Hospital of China Medical University Shenyang 110022 China; 3 Teaching Center for Basic Medical Experiment China Medical University Shenyang 110122 China; 4 Animal Science and Veterinary Medicine College Shenyang Agricultural University Shenyang 110866 China; 5School of Life Sciences China Medical University Shenyang 110122 China

**Keywords:** rolling circle amplification, stem, initiation inhibition, design principle, nucleic acid detection

## Abstract

Rolling circle amplification is a widely used biosensing technique. Although various secondary structures have been employed in RCA, their effects on RCA efficiency have seldom been reported. Here, we find that stems in circular templates can strongly inhibit RCA, and the primer-stem distance is responsible for the inhibition. Based on the results, we propose an initiation inhibition mechanism and present a design principle for a general RCA assay. Inspired by this mechanism, we further propose a new nucleic acid detection method. The results verify that this method can increase RCA detection sensitivity according to the target recycling principle. Besides DNA detection, it can also achieve single mismatch discrimination of miRNA detection after optimization. This method also shows convenient visualization detection. The initiation inhibition of RCA could be helpful for RCA applications as promising detection techniques.

## Introduction

Nucleic acid amplification techniques (NAATs) are widely used in bioassays for their fast and convenient advantages and their potential to be exploited into promising POCT (point of care testing) in IVD ( *in vitro* diagnostics). Rolling circle amplification (RCA) is an isothermal amplification technique [ 1, 2] . In RCA, circular single-stranded DNA (ssDNA) is used as a template. DNA polymerase, such as phi29, can extend the primer to produce long ssDNA along a circular template round by round. The DNA product has a repetitive sequence complementary to the circular template and can be detected by various strategies, such as fluorescence, electrochemistry, and colloidal gold [ 3‒ 5] . By virtue of its simple principle and easy operation, RCA has been used as a powerful isothermal amplification technique to detect a wide range of targets, including DNAs [6], microRNAs [7], proteins [8], small molecules [9], viruses [10], and bacteria [11].

Meanwhile, an increasing number of their special characteristics have been revealed. The nucleotide contents of circular templates have been found to affect RCA efficiency [12]. The nucleotide types of templates affect visualization detection of RCA by silver staining [13]. The template lengths are related to a sinuous phenomenon of RCA efficiency [14]. Circular templates containing nucleotide analogs can decrease RCA efficiency [15]. These findings show that the contents, lengths, sequences and modifications of circular templates can affect RCA in different aspects. They present unexpected insights into RCA and valuable guidance for RCA applications. Except contents, lengths sequences of circular templates, it has seldom been reported that the secondary structures in templates can affect RCA efficiency. Circular templates have been designed with many secondary structures as functional components, such as stems, aptamers, G4 quadruplexes and nanostructures, for various RCA application purposes [ 16‒ 19] . None of above studies have focused on how the secondary structures on circular template effect on RCA amplification rate, as well as its associated mechanisms. However, in our experiments, we found that template secondary structures such as stems can indeed inhibit RCA. These findings attracted our interest in exploring the inhibition phenomenon from the aspect of the replication mechanism.

The DNA replication process *in vivo* can be divided into initiation, elongation, and termination stages. The initiation of replication of phi29 DNA polymerase *in vivo* requires terminal protein (TP) as a primer and SSB (single-stranded DNA binding protein) and DBP (double-stranded DNA binding protein) to stabilize the template [20]. However, in RCA techniques *in vitro*, phi29 DNA polymerase initiates and elongates RCA without the help of other proteins. Although RCA initiation *in vivo* has long been clarified, phi29 DNA polymerase initiation *in vitro* in the RCA technique has seldom been explored. Similar to other DNA polymerases in replications, it requires an ssDNA template, a primer and dNTP. In terms of difference, RCA initiation *in vitro* starts from ssDNA rather than dsDNA template which is required to be unwound by denaturing or helicase *in vivo*. Therefore, the ssDNA template *in vitro* is more prone to form secondary structures than dsDNA. However, to our knowledge, the relationship between the secondary structure of the template and RCA initiation has seldom been explored.

In the present study, we used circular templates designed with stems as a secondary structure model and investigated their effects on RCA. We found that the primer-stem distance strongly affected RCA efficiency. Based on the results and structure analyses, we identified the inhibition phenomenon occurring in the initiation stage of RCA and proposed an initiation inhibition mechanism. This presented a useful principle of primer or template design for RCA techniques. In addition, by using this mechanism, we established a nucleic acid detection method, called release-initiation-recycle RCA (RIR-RCA), with an increased sensitivity by over 100-fold. This method can achieve miRNA detection and single mismatch discrimination. It is also suitable for convenient naked eye nucleic acid detection with enhanced sensitivity.

## Materials and Methods

### Materials

Oligonucleotides were purchased from Genscript Biotech Corp. (Nanjing, China). They were chemically synthesized, purified, and quantified. Oligonucleotides for preparing circular templates were modified by a 5′ phosphate group. The sequences of oligonucleotides are listed in Supplementary Table S1.

Phi29 DNA polymerase was purchased from Thermo Scientific (Waltham, USA). T4 DNA ligase and exonucleases I and III were purchased from TaKaRa Biotechnology (Dalian, China). Silica-coated magnetic beads were purchased from Luoyang Huiqing Biotechnology Co. Ltd. (Luoyang, China).

### Ligation and RCA reactions

In total, the 10 μL ligation reaction system contained 5 mM linear template, 10 mM splint oligonucleotide, 66 mM Tris-HCl, pH 7.6, 6.6 mM MgCl _2_ (magnesium chloride), 10 mM DTT (dithiothreitol), 0.1 mM ATP (adenosine triphosphate), and 1250 U/μL T4 ligase. The reaction mixture was incubated at 37°C for 30 min. Then, exonuclease I (0.625 U) and III (125 U) were added and incubated at 37°C for 30 min, followed by heating at 95°C for 10 min and cooling down to room temperature slowly. The circular template was purified by silica-coated magnetic beads according to the commercial manual. Briefly, after binding DNA to silica-coated magnetic particles using isopropanol reagents in the presence of 170 mM NaCl (sodium chloride) and magnetic separation, the binding DNA was washed three times with 70% ethanol and finally eluted with TE buffer.

The RCA reaction was carried out by using the prepared circular templates. In total, a 100 μL reaction system contained phi29 DNA polymerase (3 U), circular template (5 nM), primer (10 nM), dNTP (0.5 mM), MgOAc (magnesium acetate; 10 mM), KOAc (potassium acetate; 66 mM, pH 7.9), and SYBR Green II (1:10000). The reaction mixture was incubated at 30°C for 30 min. The fluorescence signal was monitored by a microplate reader (Infinite M200; Tecan, Seestrasse, Switzerland) every minute for at least 30 min, with an emission wavelength of 480 nm and an excitation wavelength of 520 nm.

For visualization detection, the RCA products were centrifuged by a mini-centrifugation machine (SCILOGEX S1010E, Roky Hill, USA) at 1400g for 10 min. The precipitants were visualized under ultraviolet light with the Tanon 2500R gel imaging analysis system (Tanon, Shanghai, China). Photographs were taken using an Apple iPhone XR under ultraviolet light. The precipitate was then transferred to a glass lid and further imaged by using a Hitachi SU3500 scanning electron microscope (Hitachi, Tokyo, Japan).

### Data collection and analysis

The RCA rate was calculated using fluorescence intensity as a function of time. Each experiment was repeated at least three times. Data are presented as the mean±SD. Secondary structures of templates were analyzed by using Mfold [21].

## Results and Discussion

### Primer-stem-dependent inhibition of RCA suggests a mechanism of initiation inhibition

**Figure 1:**
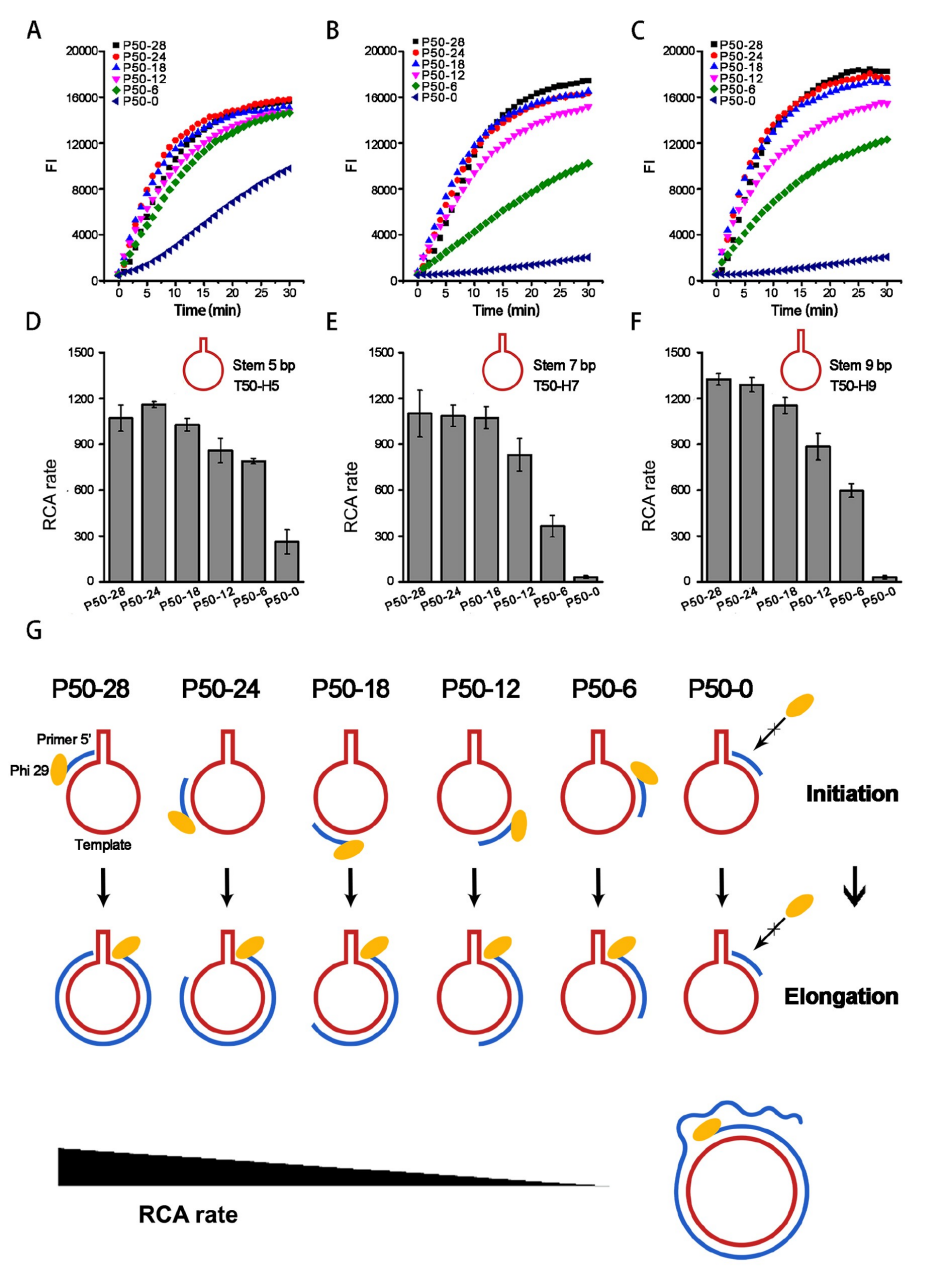
Figure 1 RCA rates affected by primer-stem distance with unchanged primer length (A‒C) Fluorescence time courses of RCA using primers P50-0/6/12/18/24/28 and templates T50-H5/H7/H9, respectively. (D‒F) RCA rates derived from (A‒C) are shown to be affected by primer binding positions. (G) Schematic of initiation inhibition to explain results in (A‒F).

In our experiments, we found that some primers have high amplification efficiency and others have low efficiency for the same RCA template. Inspired by this phenomenon, we wondered whether there are some factors related to templates and primers affecting RCA efficiency. To this end, we designed a 50-nt loop circular template with a 5-bp stem (T50-H5) and designed six primers (P50-0, P50-6, P50-12, P50-18, P50-24, and P50-28) which bind to different positions of the template loop (0 nt‒28 nt from the 3′ end of the primer to the stem). The circularization of templates was verified by electrophoresis ( Supplementary Figure S1). We found that the RCA rates of different primers were obviously different. When the primer binding position approached the stem from its 3′ end direction, the RCA was strongly inhibited ( Figure 1A,B). In addition to a template with a 5-bp stem (T50-H5), we also designed two other templates with 7-bp and 9-bp stems (T50-H7 and T50-H9, respectively) and compared these templates. We found that the effects of primer positions on RCA rates for both T50-H7 and T50-H9 were similar to those of T50-H5. Moreover, for the same primer, the longer the stem was, the lower the RCA rate was ( Figure 1C‒F).

Besides the T50 template, we also designed two other circular templates with different loop lengths and sequences. One template was designed with a 45-nt loop (T45). Five primers were prepared to bind to different positions of T45 (P45-0, P45-6, P45-12, P45-18, and P45-23). The other template was designed with a 40-nt loop (T40). Four primers were designed to bind to different positions of T40 (P40-0, P40-6, P40-12, and P40-18). Both T45 and T40 templates were designed with 5-, 7-, and 9-bp stems (T45-H5/7/9 and T40-H5/7/9 respectively; Supplementary Figures S2 and 3). For all these templates, similar inhibition effects of RCA by nearby stems were found, which verified a general phenomenon. The above results showed that the RCA efficiency was apparently affected by the primer-stem distance. When the primer-stem distance was longer than 12‒24 nt, the RCA efficiency only decreased slightly. When the distance was less than 6‒12 nt, the RCA rate was strongly decreased. Stem length was also closely related to inhibition. The inhibition order for stem length was 9 bp> 7 bp> 5 bp in general. The longer the stem was, the lower the RCA rate was.

**Figure 2:**
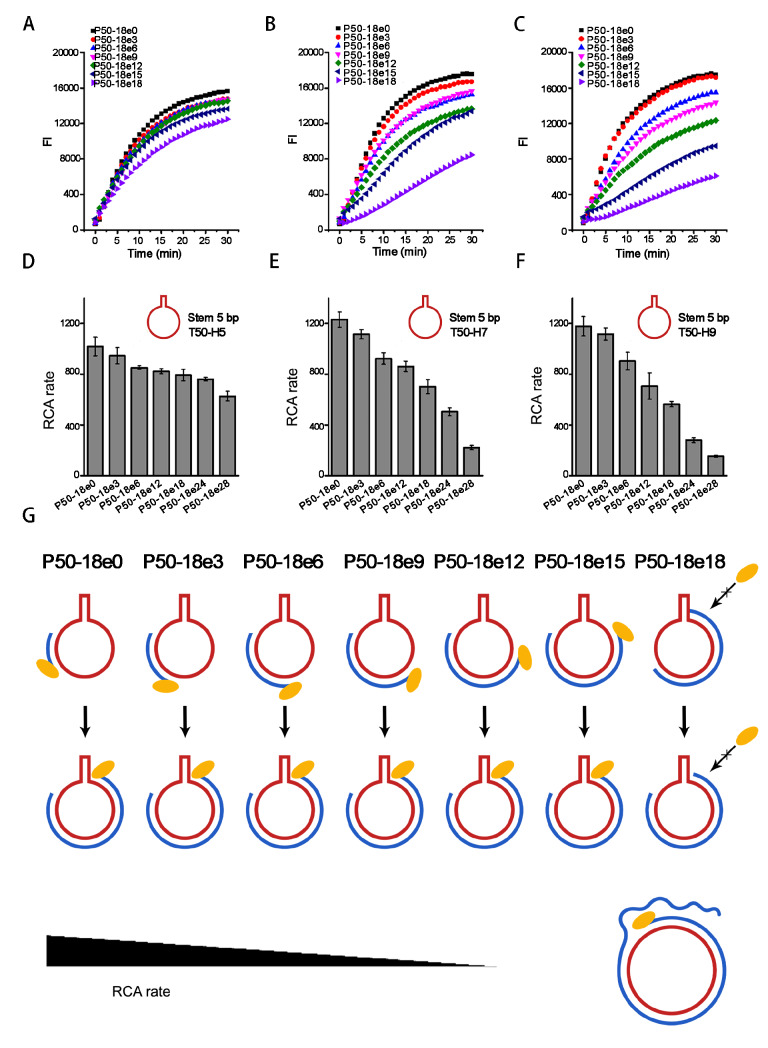
Figure 2 RCA rates affected by primer-stem distance with fixed primer 5′ end position (A‒C) Fluorescence time courses of RCA using primers P50-18e0/e3/e6/e9/e12/e15/e18 and templates T50-H5/H7/H9, respectively. (D‒F) RCA rates derived from (A-C) are shown to be affected by primer 3′ end position. (G) Schematic of initiation inhibition to explain results in (A‒F).

To further clarify the exact role of the 3′ end position rather than the 5′ end of the primer, we designed another set of primers which shared the same 5′ end position but had varied 3′ end positions. P50-18 (or P50‒18e0) was derived to be a set of primers (P50-18e3, e6, e9, e12, e15, and e18) to approach the stem by elongating its 3′ end. When primers P50‒18e3~e18 beside the stem were used, we found that the RCA efficiency was decreased accordingly. The inhibition effects of P50-18e3~e18 depended on their 3′ end distances from the stems. The shorter the distance was, the stronger the RCA inhibition was ( Figure 2A,B). These results clearly confirmed that RCA inhibition is related to the 3′ end rather than the 5′ end position of the primer. In addition, stems of 9 bp and 7 bp lengths induced stronger RCA inhibition than stems of 5 bp length ( Figure 2C‒F).

The RCA reaction begins with initiation, followed by an elongation process. In our experiments, during RCA initiation, although different primers bound to different positions of the same template, after initiation, the elongation processes were the same for the same circular template. Therefore, it is reasonable that the RCA inhibition we observed is due to the initiation stage of RCA before the primer can be extended. After initiation, phi29 DNA polymerase can break down the upcoming stem structure to continue the RCA process ( Figure 1G). In terms of primers P50-18e0~e18, P50‒18e0 can be extended to primers P50-18e3~e18 by phi29 DNA polymerase, and the extending primer during the elongation process continued without inhibition. However, when we used P50-18e0~e18 as primers, it gradually failed to initiate RCA, resulting in low RCA efficiency. This further verified that RCA inhibition came from the initiation stage rather than the primer elongation stage of RCA ( Figure 2G). When we compared Figure 1 with Figure 2, it is interesting to find that the RCA decrease of the latter changed more gradually than that of the former. This may be due to the change in primer length. The extended primer forms a rigid duplex with the template, which may force the stem in the template to be unwounded. Therefore, to some extent, it counterbalanced the rapid RCA rate decrease when the 3′ end of the primer approached the stem.

Phi29 DNA polymerase mainly includes thumb, palm, finger, TPR2 and exonuclease domains. TPR2 along with palm and thumb subdomains surround the upstream duplex. TPR2, finger, palm, and exonuclease subdomains form a special narrow tunnel with a diameter of approximately 10 Å, which can only pass the downstream single-stranded template [22]. The tunnel is necessary for phi29 DNA polymerase to unwind the downstream duplex by displacement [22]. The extended 3′ end of the primer is embraced by phi29 DNA polymerase in the upstream tunnel, which forms an angle with the downstream tunnel of the upcoming template [23]. The bend angle of the template embraced by the polymerase is approximately 90° [24]. The steric exclusion and template bending at the tunnel contribute to unwinding the downstream duplex, resulting in the displacement and processivity of the polymerase. In terms of the RCA mechanism *in vivo*, phi29 DNA polymerase requires the help of TP, SSB, and DBP to guarantee correct initiation. The synthesis of the first five nucleotides is primed by TP as initiation, and the synthesis of the sixth nucleotide will start transition from TP-priming to DNA-priming [20]. During elongation, the displacement and processivity ability can help phi29 DNA polymerase to go through complex structures in templates [25]. For RCA techniques *in vitro*, few studies have focused on the stages (initiation and elongation) of RCA. Without assistant proteins, phi29 DNA polymerase can only help itself to locate the primer’s 3′ end and dNTP at the correct active center during the initiation stage. Then, it can finally start and dash forward to overcome the secondary structure in its way by tunnel filtering and bending during the elongation stage.

**Figure 3:**
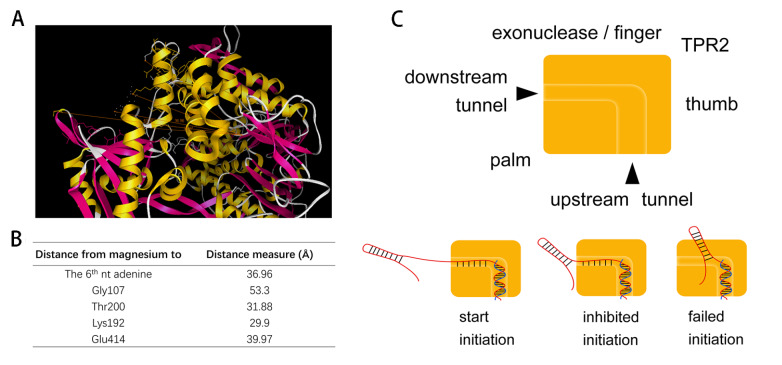
Figure 3 The length of downstream tunnel indicates the key distance from the active center to the downstream stem (A,B) Distance from magnesium in active center to the 6 th adenine and four residues located in the distal of the narrow tunnel exit. (2PYJ) (C) Schematic of the initiation inhibition explained by structure of phi29 DNA polymerase. Thumb/palm/finger/exonuclease/TPR2 domains and upstream/downstream tunnels in polymerase structure are indicated in the upper panel. The positions and effects of stem on polymerase are shown in the lower panel.

Our results showed that stem is not a problem during the elongation stage but a problem for nearby initiation primers, which clearly identified an initiation inhibition of RCA. This also explained why the 3′ end rather than the 5′ end position of the primer counted in RCA inhibition. In this case, the stem interfered with phi29 DNA polymerase to locate the primer’s 3′ end to initiate RCA. Moreover, this interference was related to the length of the stem, which suggested steric hindrance of the stem. Longer stems are more thermostable than shorter stems (ΔG=‒3, ‒7, and ‒10 kcal/mol for 3-, 7-, and 9-bp stems, respectively). It has been found that phi29 DNA polymerase spends a longer time at high GC content stems because of the relatively stable stem structure [23]. This is consistent with our results that long or stable stems strongly inhibited RCA. How to explain the effects of the length of primer-stem distance on RCA initiation inhibition? From the tertiary structure of phi29 polymerase (2PYJ), we found that after right angle bending, the downstream single-stranded template passed approximately 6 nt long through the tunnel to pass the polymerase coverage. The terminal nucleotide of the single-stranded template was the sixth nucleotide downstream of the primer’s 3′ end (2PYJ). It just passed through the tunnel. We analyzed the distance from the active center (magnesium ion) to the distal surface (Gly107) of the polymerase in the template downstream direction through the tunnel. The distance was 5.33 nm, which was approximately the full length of 6 nt ssDNA. If 6 nt ssDNA zigzags its way, it may just go through the tunnel (approximately more than 3 nm distance) ( Figure 3A,B). Therefore, the absence of at least a 6 nt long downstream single-stranded template is very important for locating the primer’s 3′ end at the active center. This is consistent with our results that the primer-stem distance change from 0 to 6 nt presents the most obvious change in RCA rate. Interestingly, for RCA initiation *in vivo*, approximately six nucleotides incorporated in the upstream tunnel triggered the dissociation of TP as a transition from initiation to elongation mode [26].

Based on our results and analyses, we proposed an initiation inhibition mechanism. The stem in the circular template in front of the primer’s 3′ end (especially less than 6 nt) inhibits phi29 DNA polymerase from accessing the primer’s 3′ end by steric exclusion; therefore, RCA cannot be initiated by the polymerase. If the stem maintains a distance of more than 12‒24 nt from the primer’s 3′ end, phi29 DNA polymerase can easily access and locate the primer’s 3′ end to the active center to start polymerization ( Figure 3C). After RCA initiation, polymerized dNTPs fuel phi29 DNA polymerase to extend the primer to process along the downstream template through its molecular tunnel and turn its way to unwind the downstream stem with the help of its displacement ability. Besides phi29 DNA polymerase, Bst DNA polymerase is also used in RCA but at higher temperatures (approximately 60°C). The lower processivity of Bst DNA polymerase suggests its relatively loose DNA holding compared with phi29 DNA polymerase [15]. Moreover, the secondary structures in templates are unstable at high reaction temperatures. Therefore, the initiation inhibition of RCA is more significant for phi29 rather than Bst DNA polymerase in theory.

This finding also suggests a design principle for RCA. In the design of primers, care should be taken when there is a secondary structure in RCA template, except intentionally implement this initiation inhibition. The downstream secondary structure less than 12 nt (especially 6 nt) from the primer’s 3′ end should be carefully checked to ensure that no stem is longer than 5 bp. Considering some reported factors affecting RCA efficiency, we summarized the suggested design principles of templates and primers for RCA assays ( Table 1). It should be noted that the table does not include the special amplification strategy design used in RCA derivative techniques, such as HRCA (hyperbranched RCA), C2C-RCA (circle to circle RCA), and PG-RCA (primer generation RCA) [ 27‒ 29] , or enhancer molecules to increase RCA efficiency, such as SSB (single strand DNA-binding protein) and PEG (polyethylene glycol) [ 30, 31] .

**Table:** **Table 1** Design principles summarized from reports to increase RCA efficiency

Suggested design principle (phi29)	Mechanism	Reference
Periodic maxima (odd helical half turns of DNA) and shorter rings	Fraying during extension	[14]
AC rich nucleotides contents	AC preference for phi29 polymerase	[12]
PS or LNA modified rather than 2′-O-methyl modified	Inhibit polymerase during extension	[15]
No stem within 12 nt downstream primer 3′ end	Inhibit polymerase from initiation	This work

### The release-initiation-recycle RCA strategy can be used for nucleic acid detection

**Figure 4:**
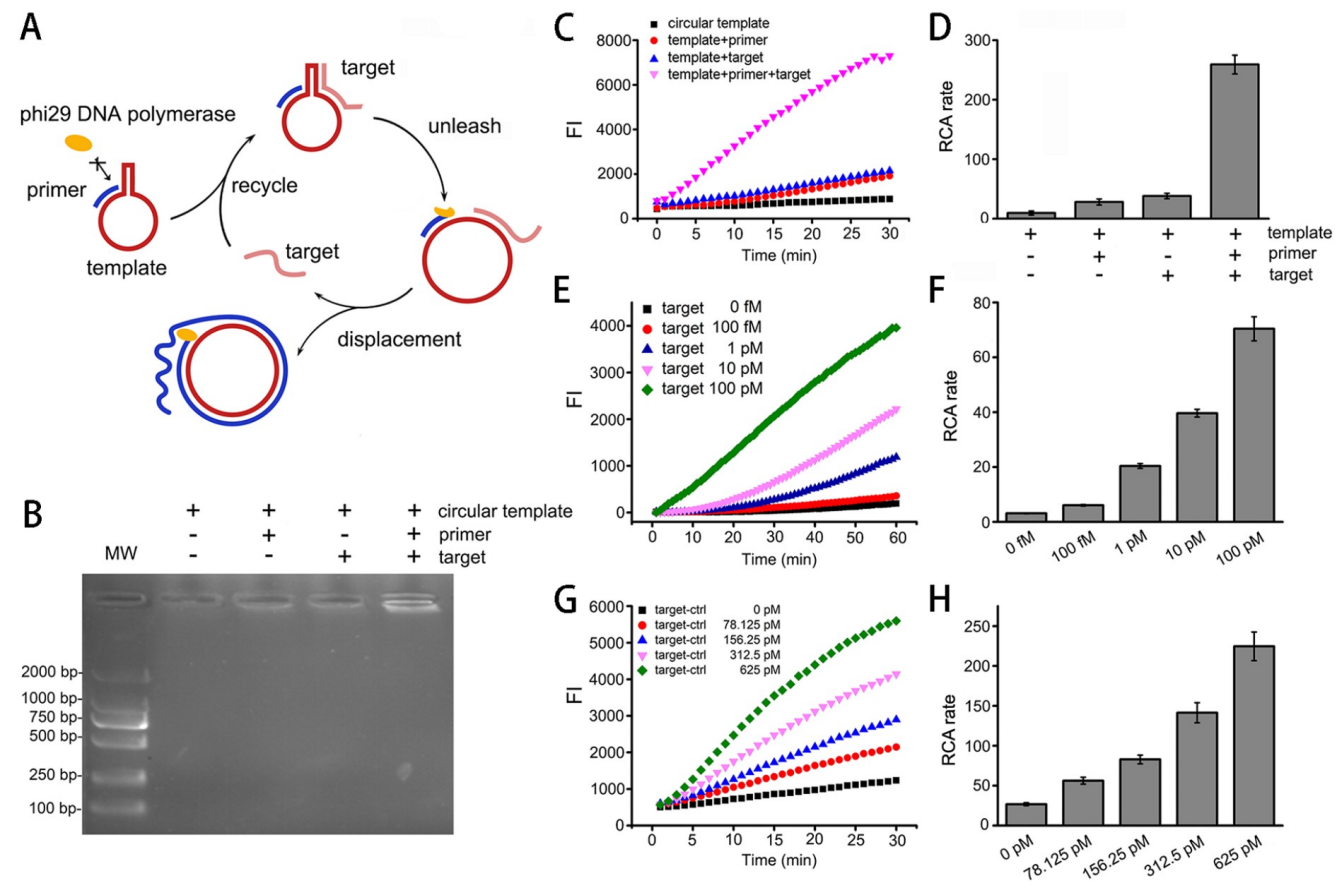
Figure 4 Proof-of-concept and sensitivity of RIR-RCA (A) Schematic of RIR-RCA. (B) Proof-of-concept of RIR-RCA verified by agarose electrophoresis. (C,D) Proof-of-concept of RIR-RCA verified by microplate reader fluorescence detection (C) and RCA rate (D). (E,F) Serial diluted targets detected by RIR-RCA fluorescence time course (E) and RCA rate (F). (G,H) Diluted targets detected by normal RCA control fluorescence time course (G) and RCA rate (H) with the same template and experimental condition as RIR-RCA but using target-control as primer.

Although initiation inhibition is unfavorable for RCA efficiency, we wondered if it can be employed in nucleic acid detection. Based on the initiation inhibition principle, a nucleic acid detection method is proposed here. We used single-stranded nucleic acids as targets to hybridize the stem on the other side of the primer to unleash initiation inhibition by opening the stem. This triggers the primer’s extension, which can displace and free the bound target in turn, resulting in another cycle of target hybridization, unleashing and displacement. Therefore, stem opening, primer extension, and target displacement can form a self-recycling amplification process. We call this strategy release-initiation-recycle RCA (RIR-RCA). ( Figure 4A)

To verify the proof-of-concept of RIR-RCA, we designed an ssDNA target which is complementary to the other side of the stem and to the adjacent loop. To avoid the extension of the target itself, we elongated the 20-nt noncomplementary sequence to the 3′ end of the target. In theory, RCA occurs only in the presence of template, primer, and the target. The results showed that the target indeed recovered the RCA rate. In comparison with the only circular template control, the addition of the primer or the target only triggered a slight increase in RCA fluorescence, which suggested a leashed primer by the stem of the template. The addition of both the target and the primer to the template strongly increased the RCA rate, which indicated the unleashed primer and the released RCA initiation by the target. This successfully verified the RIR-RCA concept ( Figure 4B‒D). According to the principle of RIR-RCA, one recycled target can trigger templates to unleash primers many times. However, for normal RCA, one target can only initiate one circular template. Therefore, the sensitivity of this method is theoretically supposed to be much higher than that of normal RCA. We explored its sensitivity by detecting a series of diluted targets by RIR-RCA and normal RCA. We found that targets as low as 0.1 pM can be identified by RIR-RCA in our experiments. In comparison with normal RCA, the sensitivity of RIR-RCA was increased by more than 100- *fold*. This further verified the effectiveness of the RIR principle ( Figure 4E‒H).

Specificity is a significant issue, especially for nucleic acid detection, such as SNP or microRNA assays. The hybridization sensing mode of RIR-RCA has the advantage of detecting both ssDNA and RNA. MicroRNAs are important nucleic acid targets in many cases [32]. They play important roles in biological processes and gene expression regulation [33]. Some of them are closely related to diseases and are potential biomarkers of diseases [34]. Because of their short lengths and high similarity between family members, the discrimination of miRNA is difficult [35]. Herein, we used miRNA detection to test the single mismatch discrimination ability of RIR-RCA.

**Figure 5:**
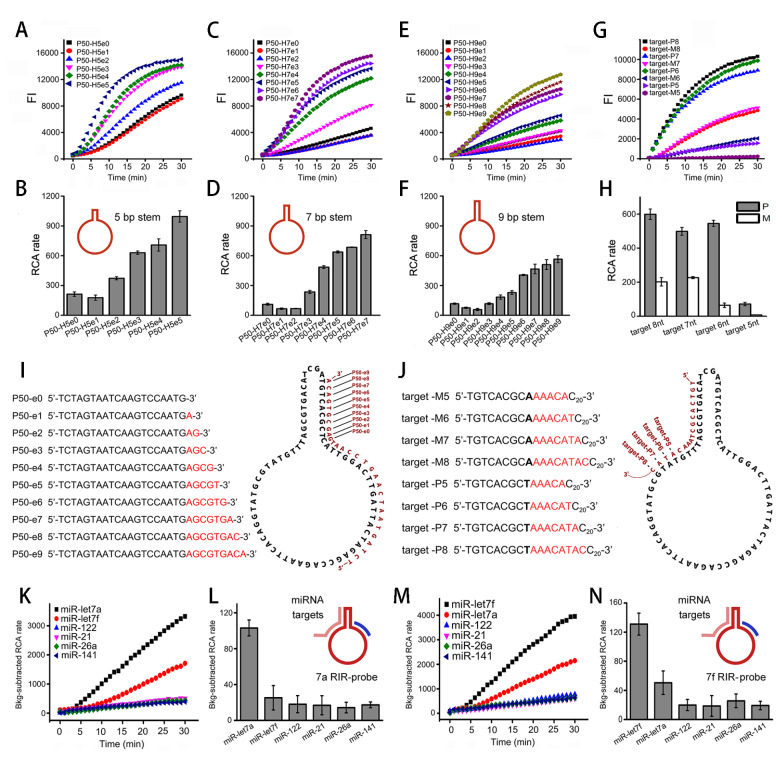
Figure 5 Specific discrimination of single nucleotide mismatch by RIR-RCA (A‒F) Optimization of stem-hybridization length to open the stem verified by fluorescence time course (A‒C) and RCA rate (D‒F). (G,H) Optimization of toehold length for specificity discrimination verified by fluorescence time course (G) and RCA rate (H). (I) Design schematic for (A‒F). Primers P50-e0/e1/e2/e3/e4/e5/e6/e7/e8/e9 were used. (J) Design schematic for (G,H). Target-M5/6/7/8 for mismatch and target-P5/6/7/8 for perfect match were used. (K,L) Let-7a discrimination from other five miRNAs by let-7a RIR-probe verified by fluorescence time course (K) and RCA rate (L). (M,N) Let-7f discrimination from other five miRNAs by let-7f RIR-probe verified by fluorescence time course (M) and RCA rate (N).

Stem opening is affected by hybridization length and stem length. To achieve single mismatch discrimination, we first optimized the hybridization reaction by extending the primer 3′ end into the stem in the template. We chose the primer P50-0 as a model, which bound to its 3′ end adjacent to the stem. We prepared three sets of primers (P50-H5e0~e5, P50-H7e0~e7, and P50-H9e0~e9) which were derived from elongating the primer’s 3′ end into three stems (H5, H7, and H9). The elongated part was complementary to the stem sequence and was supposed to open the stem to some extent. With the increasing length of the primer, the inhibited RCA rate was recovered due to the opened stem by primer hybridization. When the stem was fully complemented by the elongated primer, the strongest RCA rate was obtained. Moreover, the shorter the stem was, the higher the recovered RCA rate was. ( Figure 5A‒F,I) The RCA initiation was triggered by the hybridization of the target with the stem and the adjacent loop as a toehold. After optimization of the hybridization length and stem length, we optimized the toehold length of hybridization. We prepared a set of perfectly matched targets and their mismatched counterparts to explore the effects of toehold length on the ability of RIR-RCA to discriminate a single mismatch. The results showed that with the increase of toehold length, the fluorescent signal of the mismatched target was increased. When we decreased the toehold length, the fluorescent signal of the matched target was decreased. We found that 6 nt length presented the strongest ability to discriminate a single mismatch ( Figure 5G,H,J).

By using the optimized conditions, we used microRNA hsa-let-7a-5p and hsa-let-7f-5p of the hsa-let-7 family as examples to test the specificity of RIR-RCA. They have only one point mutation difference between their sequences. Two circular templates, T50-let7a and T50-let7f, were designed for the miRNA targets let-7a and let-7f, respectively. In addition, we also selected four other miRNAs (miR-122, miR-21, miR-26a, and miR-141) for specificity verification. The circularization of templates was verified by electrophoresis ( Supplementary Figure S4). The results of T50-let7a detection are shown in Figure 5K,L. Only let-7a generated a strong fluorescence signal. The fluorescence value induced by let-7f was lower than that induced by let-7a, and the other four miRNAs at the same concentration only triggered a slight increase in RCA fluorescence. We also conducted miRNA detection using T50-let7f. Only let-7f induced a strong RCA rate, while let-7a presented a slight fluorescence increase ( Figure 5M,N). The discrimination of let-7a and let-7f by T50-let7a was more apparent than that by T50-let7f. This might be due to the G-T mismatch by using T50-let7f. Therefore, the above results verified that the RIR-RCA method can even distinguish single nucleotide for miRNA detection.

**Figure 6:**
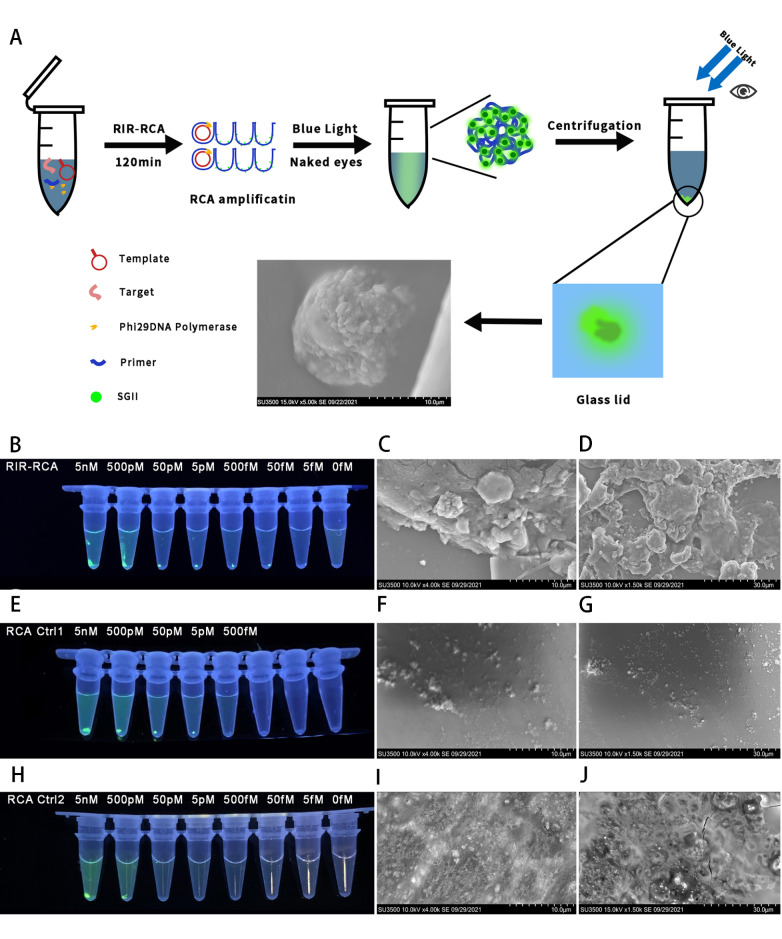
Figure 6 Naked eye visualization detection by RIR-RCA (A) Illustration of workflow for visualization detection by RIR-RCA. The sensitivity of visualization detection under ultraviolet for RIR-RCA (B), common stem RCA (E), and common non-stem RCA (H). Scanning electron microscopy images of product DNA blot of 10 pM target RIR-RCA (C,D), common stem RCA (F,G), and common non-stem RCA (I,J).

RCA is an isothermal amplification suitable for POCT development free of expensive equipment. We found that RIR-RCA can achieve convenient detection over common RCA by naked-eye visualization under ultraviolet light with the help of centrifugation ( Figure 6A). The direct visualization of RCA products under ultraviolet light was difficult, especially for low product concentrations, while the RIR-RCA products precipitated easily, resulting in an enhanced visualization efficiency. The concentrated RCA products were even easier to identify by the naked eyes on various carriers, such as tubes, paper and glass ( Supplementary Figure S5). By comparing RIR-RCA with common RCA using centrifugation-assisted visualization, we obtained consistent results with those of microplate reader detection. By concentrating the dye-binding RCA products, RIR-RCA can detect targets as low as the femtomolar level, which is more sensitive than common RCA detection. The common nonstem RCA was less sensitive than common stem RCA for visualization, which might suggest a stem-assisted precipitation of RCA products during centrifugation ( Figure 6B,E,H). The precipitant was further imaged by scanning electron microscopy (SEM) to confirm the RIR-RCA products ( Figure 6C,D,F,G,I,J). We can see the nanostructure of RCA hydrogel products in the RIR-RCA samples. The hydrogel formation of RCA products is dependent on amplification time [36]. Here, the self-assembled hydrogel was rapidly constructed after 2 h of RCA followed by only 10 min of centrifugation. The inorganic magnesium pyrophosphate can be coprecipitated as nanocrystalline [37]. Fluorescent dyes have also been found to help the precipitation of RCA products through London forces [38]. A cross-link DNA network can be constructed by a complementary RCA product [39]. In our experiments, interstrand stem hybridization may contribute to the formation of the precipitant with the help of inorganic nanocrystals. The dyes can be easily concentrated through RCA stem product precipitation. Therefore, it provides a convenient visualization method with enhanced sensitivity. In comparison with microplate reader detection, visual detection is suggested for convenient semiquantitative detection rather than quantitative detection.

## Conclusions

In summary, we found that the stem in the circular template beside the primer 3′ end inhibited RCA, especially in the case of primer-stem distance less than 6-12 nt. We proposed an initiation inhibition mechanism and suggested general design principles for RCA. Furthermore, based on this principle, we proposed a nucleic acid detection method RIR-RCA by using a target to unleash initiation inhibition. The RIR-RCA has several advantages for nucleic acid detection: (1) the sensitivity is obviously enhanced; (2) the hybridization sensing mode is suitable for not only ssDNA but also RNA; (3) it can achieve single base mismatch discrimination; and (4) it can achieve convenient and sensitive visualization detection after centrifugation. In future studies, whether initiation inhibition exists and the role played by other secondary structures and other polymerases remain to be explored.

## Supporting information

22475supplementary_data

## Supplementary Data

Supplementary data is available at *Acta Biochimica et Biophysica Sinica* online.
