# Supplementary material for: Revealing an initiation inhibition of RCA and its application in nucleic acid detection: Initiation inhibition of RCA to detect nucleic acids
Source: Acta Biochim Biophys Sin (Shanghai). 2023 Apr 20;55(4):672–82. doi: 10.3724/abbs.2023070 (PMC10195145; doi:10.3724/abbs.2023070)
Supplement: 22475supplementary_data [file 22475supplementary_data.pdf]

Supplementary Table S1. Oligonucleotides used in experiments

Name	Sequence
T40-H5	5′-P-TGATTACTAGAGCCAGAATTGGTGTCGACACCATTAGGTATGCGTATGTTTA-3′
T40-H7	5′-P-TGATTACTAGAGCCAGAATTGCGTCATCGATGACGCATTAGGTATGCGTATGTTTA-3′
T40-H9	5′-P-TGATTACTAGAGCCAGAATTGCGTGACATCGATGTCACGCATTAGGTATGCGTATGTTTA-3′
Splint-T40	5′-CTAGTAATCATAAACATACG-3′
P40-0	5′-ATAAACATACGCATACCTAA-3′
P40-6	5′- GTAATCATAAACATACGCAT-3′
P40-12	5′- GCTCTAGTAATCATAAACAT-3′
P40-18	5′- ATTCTGGCTCTAGTAATCAT-3′
T45-H5	5′-P-TGATTACTAGAGCCAGAATTGGTGTCGACACCATACAAATAGGTATGCGTATGTTTA-3′
T45-H7	5′-P-TGATTACTAGAGCCAGAATTGCGTCATCGATGACGCATACAAATAGGTATGCGTATGTTTA-3′
T45-H9	5′-P-TGATTACTAGAGCCAGAATTGCGTGACATCGATGTCACGCATACAAATAGGTATGCGTATGTTTA-3′
P45-0	5′-CATACGCATACCTATTTGTA-3′
P45-6	5′-CATAAACATACGCATACCTA-3′
P45-12	5′-AGTAATCATAAACATACGCA-3′
P45-18	5′-GGCTCTAGTAATCATAAACA-3′
P45-23	5′-ATTCTGGCTCTAGTAATCAT-3′
T50-H5	5′-P-GAATTCACAGGTATGCGTATGTTTAGGTGTCGACACCTCATTGGACTTGATTACTAGAGCCA-3′
T50-H7	5′-P-GAATTCACAGGTATGCGTATGTTTAGCGTCATCGATGACGCTCATTGGACTTGATTACTAGAGCCA -3′
T50-H9	5′-P-GAATTCACAGGTATGCGTATGTTTAGCGTGACATCGATGTCACGCTCATTGGACTTGATTACTAGAGCCA -3′
Splint-T50	5′-CTGTGAATTCTGGCTCTAGT-3′
P50-0	5′-TCTAGTAATCAAGTCCAATG-3′
P50-6	5′-TCTGGCTCTAGTAATCAAGT-3′
P50-12	5′-GTGAATTCTGGCTCTAGTAA-3′
P50-18	5′-ATACCTGTGAATTCTGGCTC-3′
P50-24	5′-ATACGCATACCTGTGAATTC-3′
P50-28	5′-AAACATACGCATACCTGTGA-3′
P50-18e3	5′-ATACCTGTGAATTCTGGCTCTAG-3′
P50-18e6	5′-ATACCTGTGAATTCTGGCTCTAGTAA-3′
P50-18e9	5′-ATACCTGTGAATTCTGGCTCTAGTAATCA-3′
P50-18e12	5′-ATACCTGTGAATTCTGGCTCTAGTAATCAAGT-3′
P50-18e15	5′-ATACCTGTGAATTCTGGCTCTAGTAATCAAGTCCA-3′
P50-18e18	5′-ATACCTGTGAATTCTGGCTCTAGTAATCAAGTCCAATG-3′
P50-H5e1	5′-TCTAGTAATCAAGTCCAATGA-3′

P50-H5e2	5′-TCTAGTAATCAAGTCCAATGAG-3′
P50-H5e3	5′-TCTAGTAATCAAGTCCAATGAGG-3′
P50-H5e4	5′-TCTAGTAATCAAGTCCAATGAGGT-3′
P50-H5e5	5′-TCTAGTAATCAAGTCCAATGAGGTG-3′
P50-H7e1	5′-TCTAGTAATCAAGTCCAATGA-3′
P50-H7e2	5′-TCTAGTAATCAAGTCCAATGAG-3′
P50-H7e3	5′-TCTAGTAATCAAGTCCAATGAGC-3′
P50-H7e4	5′-TCTAGTAATCAAGTCCAATGAGCG-3′
P50-H7e5	5′-TCTAGTAATCAAGTCCAATGAGCGT-3′
P50-H7e6	5′-TCTAGTAATCAAGTCCAATGAGCGTC-3′
P50-H7e7	5′-TCTAGTAATCAAGTCCAATGAGCGTCA-3′
P50-H9e1	5′-TCTAGTAATCAAGTCCAATGA-3′
P50-H9e2	5′-TCTAGTAATCAAGTCCAATGAG-3′
P50-H9e3	5′-TCTAGTAATCAAGTCCAATGAGC-3′
P50-H9e4	5′-TCTAGTAATCAAGTCCAATGAGCG-3′
P50-H9e5	5′-TCTAGTAATCAAGTCCAATGAGCGT-3′
P50-H9e6	5′-TCTAGTAATCAAGTCCAATGAGCGTG-3′
P50-H9e7	5′-TCTAGTAATCAAGTCCAATGAGCGTGA-3′
P50-H9e8	5′-TCTAGTAATCAAGTCCAATGAGCGTGAC-3′
P50-H9e9	5′-TCTAGTAATCAAGTCCAATGAGCGTGACA-3′
target	5′-TGTCACGCTAAACATACGCATACCTGTGACCCCCCCCCCCCCCCCCCCC-3′
target-ctrl	5′-TGTCACGCTAAACATACGCATACCTGTGA-3′
target-M5	5′-TGTCACGCAAAACACCCCCCCCCCCCCCCCCCCC-3′
target-M6	5′-TGTCACGCAAAACATCCCCCCCCCCCCCCCCCCCC-3′
target-M7	5′-TGTCACGCAAAACATACCCCCCCCCCCCCCCCCCCC-3′
target-M8	5′-TGTCACGCAAAACATACCCCCCCCCCCCCCCCCCCCC-3′
target-P5	5′-TGTCACGCTAAACACCCCCCCCCCCCCCCCCCCC-3′
target-P6	5′-TGTCACGCTAAACATCCCCCCCCCCCCCCCCCCCC-3′
target-P7	5′-TGTCACGCTAAACATACCCCCCCCCCCCCCCCCCCC-3′
target-P8	5′-TGTCACGCTAAACATACCCCCCCCCCCCCCCCCCCCC-3′
miR-let7a	5′-UGAGGUAGUAGGUUGUAUAGUU-3′
miR-let7f	5′-UGAGGUAGUAGAUUGUAUAGUU-3′
T50-let7a	5′-GAATTCACAGGTATTTGAATACAACCTACTACCTCGAGGTAGTAGGCATTGGACTTGATTACTAGAGCCA-3′
T50-let7f	5′-GAATTCACAGGTATTTGAATACAATCTACTACCTCGAGGTAGTAGACATTGGACTTGATTACTAGAGCCA-3′
miR-122	5′-UGGAGUGUGACAAUGGUGUUUG-3′
miR-21	5′-UAGCUUAUCAGACUGAUGUUGA-3′
miR-26a	5′-UUCAAGUAAUCCAGGAUAGGCU-3′
miR-141	5′-CAUCUUCCAGUACAGUGUUGGA-3′

Splint hybridization sequence is marked by a wave line. Stem domain is underlined.

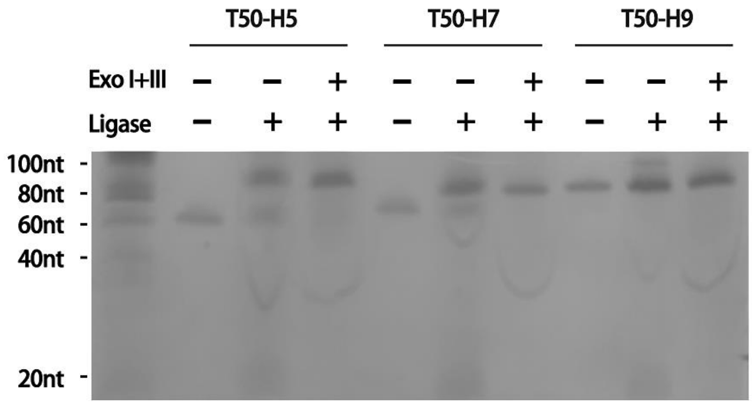

Supplementary Figure S1. Circularization of linear templates T50-H5/H7/H9 verified by denaturing polyacrylamide gel electrophoresis After ligation, the migration of circular template became slower. After addition of exonucleases, the linear oligonucleotides were digested.

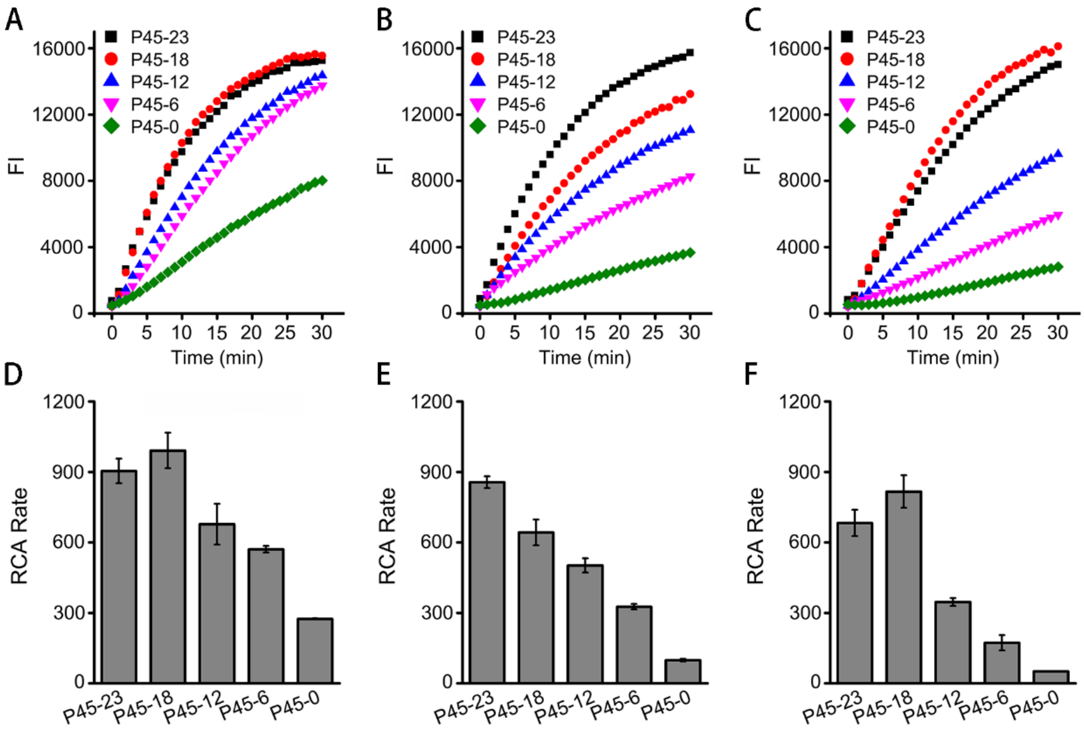

Supplementary Figure S2. Effects of primer-stem distance on RCA rate for T45 template Fluorescence time course (A) and RCA rate (D) of different primers for T45-H5 template with a 5-bp stem. Fluorescence time course (B) and RCA rate (E) of different primers for T45-H7 template with a 7-bp stem. Fluorescence time course (C) and RCA rate (F) of different primers for T45-H9 template with a 9-bp stem. Primers P45-0/6/12/18/23 were used respectively.

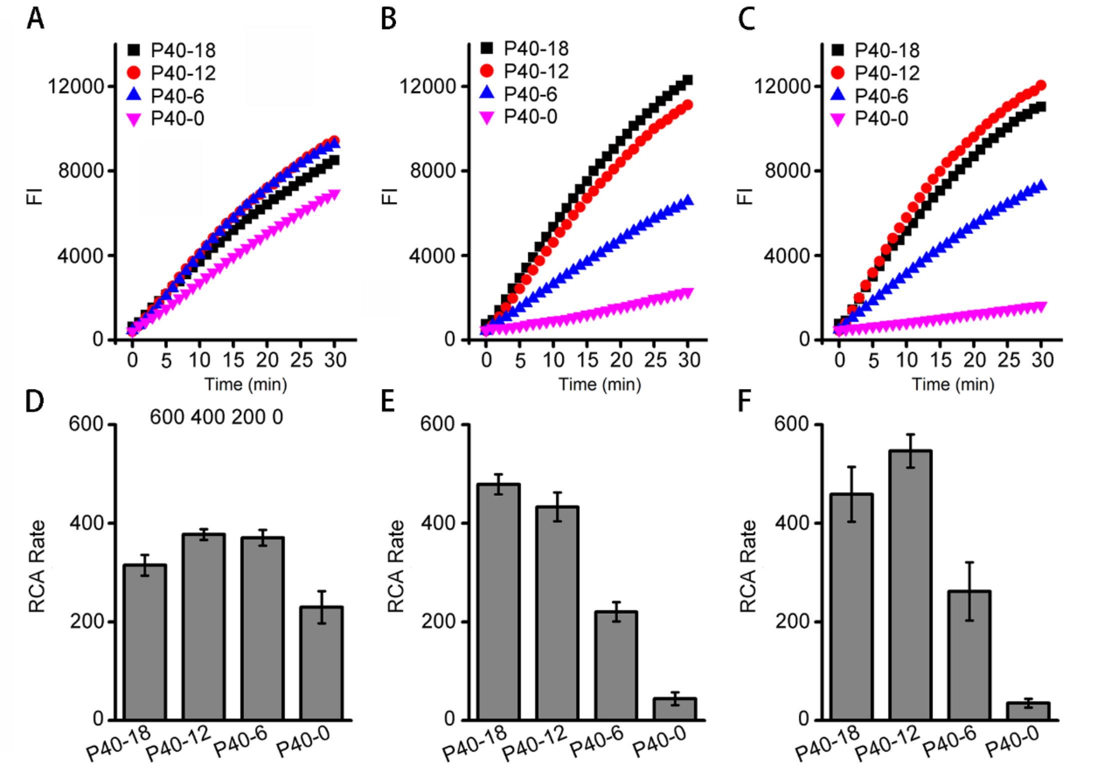

Supplementary Figure S3. Effects of primer-stem distance on RCA rate for T40 template Fluorescence time curve (A) and RCA rate (D) of different primers for T40-H5 template with a 5-bp stem. Fluorescence time curve (B) and RCA rate (E) of different primers for T40-H7 template with a 7-bp stem. Fluorescence time curve (C) and RCA rate (F) of different primers for T40-H9 template with a 9-bp stem. Primers P40-0/6/12/18 were used respectively.

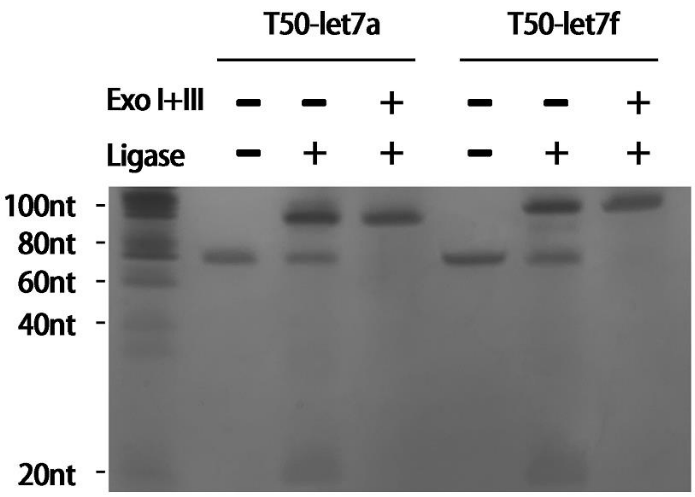

Supplementary Figure S4. Circularization of linear templates T50-let7a/7f verified by denaturing polyacrylamide gel electrophoresis After ligation, the migration of circular template became slower. After addition of exonucleases, the linear oligonucleotides were digested.

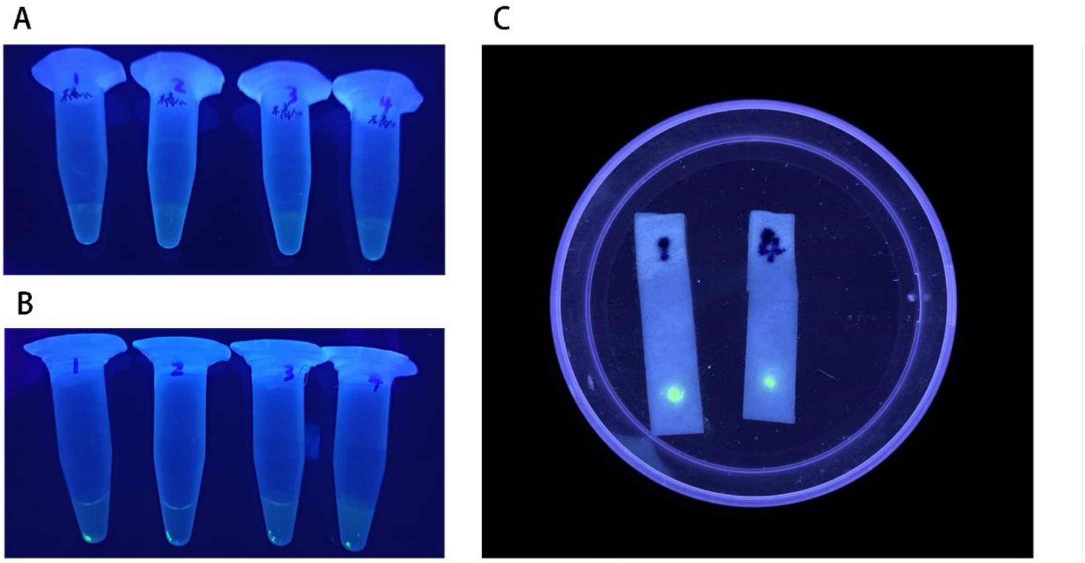

Supplementary Figure S5. Centrifugation-assisted visualization of RIR-RCA Quadruplet samples of RIR-RCA (50 pM target) after 2 h of incubation before centrifugation (A), and after centrifugation (B). Dim samples become easily identified by fluorescent blot at the bottom. (C) DNA sediment can be transferred and blotted to other carriers, such as paper and glass. The samples on paper can be stored for a long time and suitable for recording.
